# Oxidative Stress and Alcohol-Related Hepatitis: A Role for Future Therapies

**DOI:** 10.3390/antiox15040493

**Published:** 2026-04-16

**Authors:** Francesca D’Arcangelo, Neil Rajoriya, Patricia F. Lalor

**Affiliations:** 1 The Liver Unit Birmingham, University Hospitals Birmingham NHS Foundation Trust, Birmingham B15 2GW, UKneil.rajoriya@uhb.nhs.uk (N.R.); 2 Centre for Liver and Gastroenterology Research and NIHR Birmingham Biomedical Research Centre, College of Medicine and Health, University of Birmingham, Birmingham B15 2GW, UK

**Keywords:** alcohol, alcohol use disorder, reactive oxygen species, antioxidant defence, alcohol hepatitis

## Abstract

Alcohol-associated Hepatitis (AH) is a rare acute injury caused by alcohol consumption, which can lead to one of the most severe manifestations of liver disease. It is part of the alcohol-related liver diseases (ArLD) spectrum, which represents a major global health burden, with oxidative stress and inflammation serving as central, interconnected pathogenic mechanisms. Chronic alcohol (ethanol) consumption induces hepatic reactive oxygen species (ROS) generation through multiple pathways, including cytochrome P450 2E1 (CYP2E1) induction, mitochondrial dysfunction, and NADPH oxidase activation. These oxidative insults trigger a cascade of cellular damage encompassing lipid peroxidation, protein adduct formation, DNA damage, and endoplasmic reticulum stress, ultimately leading to hepatocyte dysfunction and multiple forms of cell death, including apoptosis, necroptosis, pyroptosis, and ferroptosis. The inflammatory response, orchestrated primarily by Kupffer cells and infiltrating neutrophils through Toll-like receptor (TLR) signalling and inflammasome activation, not only amplifies hepatic injury but also promotes fibrogenesis through hepatic stellate cell activation. Neutrophils, characterised by elevated lipocalin-2 expression and spontaneous NETosis in AH, exhibit a paradoxical role by driving both tissue damage and repair. Current therapeutic strategies include corticosteroids, which remain the first-line treatment for severe AH, while emerging therapies targeting the gut–liver axis, hepatic regeneration, and specific molecular targets show promise in clinical trials. This review comprehensively examines the molecular crosstalk between oxidative stress and inflammation in the pathogenesis of AH to highlight current and investigational therapeutic approaches targeting these interconnected pathways.

## 1. Introduction

Alcohol-associated Hepatitis (AH), previously termed Alcoholic Hepatitis, represents one of the most challenging disease manifestations within the spectrum of alcohol-related liver diseases (ArLD). This is due to its acute presentation, challenges in confirmatory diagnosis and disease severity. The most acute florid severe form is characterised by high short-term mortality up to 50% within three months of diagnosis [1]. Recent evidence has highlighted the interplay between hepatocyte injury and oxidative stress, systemic inflammatory response and gut–liver axis dysfunction [2,3,4,5,6,7,8,9,10,11] in AH. Currently, European and American clinical practice guidelines recommend corticosteroid therapy in selected patients with a Maddrey’s Discriminant Function (MDF) score > 32 or a Model for End-Stage Liver Disease (MELD) score > 20, with recent evidence reporting the greatest benefit for those with MELD scores between 25 and 29 [12,13,14,15]. Corticosteroids are, however, not associated with long-term survival benefit [16] and in eligible cases, liver transplantation remains the only therapeutic option for patients unresponsive to medical treatment [14,17,18]. The aim of this review is to analyse the role of oxidative stress in the pathophysiology of AH and to highlight the recent therapeutic innovations proposed to treat this life-threatening condition.

## 2. Alcohol-Associated Hepatitis: Definition, Prevalence and Natural History

**Figure 1:**
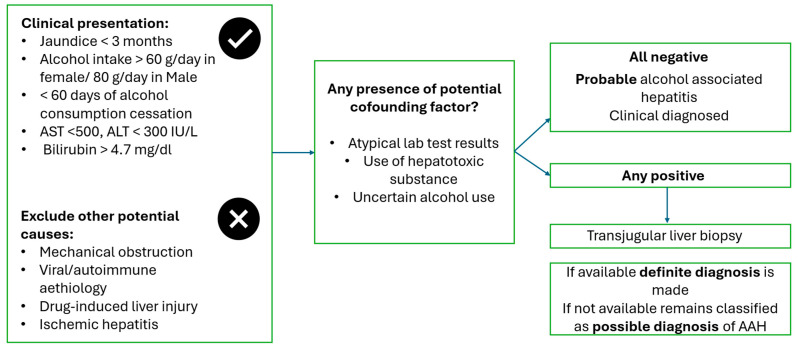
Diagnostic flowchart of acute alcoholic hepatitis.

The definition of AH has recently been revised by the National Institute on Alcohol Abuse and Alcoholism (NIAAA)–AH Consortium, which has categorised AH into definite, probable, and possible diagnosis [19]. AH is defined as a clinical syndrome characterised by the association of new onset of jaundice and alteration of liver blood tests (AST < 500 IU/mL, ALT < 300 IU/mL within three months from clinical presentation and with total serum bilirubin > 80 μmol) in female/male patients with more than 60/80 g of daily alcohol use with less of 60 days of alcohol cessation [16]. Other potential causes of liver disease must be excluded, and liver biopsy may be performed in the presence of potential confounding factors, depending on local practice. A definitive diagnosis can be established with histologic confirmation (Figure 1), although the findings of steatohepatitis can be similar to those described in metabolic-associated steatohepatitis (MASH). Parker et al. [20] reported on a systematic review from 7528 biopsy-proven ArLD participants, of which 25% had steatohepatitis. These patients had a higher mortality when compared to those with simple steatosis alone due to alcohol. Although no single hallmark has been identified, features such as megamitochondria, pericellular fibrosis, macrovesicular steatosis, cholestasis and Mallory Denk bodies are more frequently observed in patients with AH than in MASH [21,22]. Thus, whilst helpful, histological interpretation should always be integrated with the clinical context. The prevalence of AH is equally variable across territories [13] but is increasing in line with alcohol consumption. Incidence is estimated to have increased during the COVID pandemic and is now more common among female and young patients [23,24]. Data from Denmark showed a rise in AH incidence in both men and women, with the increase being particularly pronounced among middle-aged female patients [25]. Similarly, UK data from the reported an increase of 31% in the number of deaths related to alcohol use from 2019 to 2022 [26]. These data are in line with those from the United States, where AH hospital admissions rose from 0.66% of total hospital admissions in 2002 to 0.83% in 2010 [27].

The clinical diagnosis is particularly challenging, as the disease manifestation in the context of sustained alcohol exposure is clinically similar to acute-on-chronic liver failure triggered by excessive alcohol consumption. In fact, real-world data from cohorts of patients transplanted for severe acute alcoholic hepatitis have revealed the presence of previously unrecognised cirrhosis in explanted livers in both American and European cohorts [28,29,30].

Hence, alcohol-related liver disease in general and alcohol-related hepatitis and ACLF caused by excess alcohol intake account for a significant proportion of global liver morbidity and mortality, which are linked to changes in patterns of alcohol intake.

## 3. Oxidative Stress Injury in Liver Diseases

**Figure 2:**
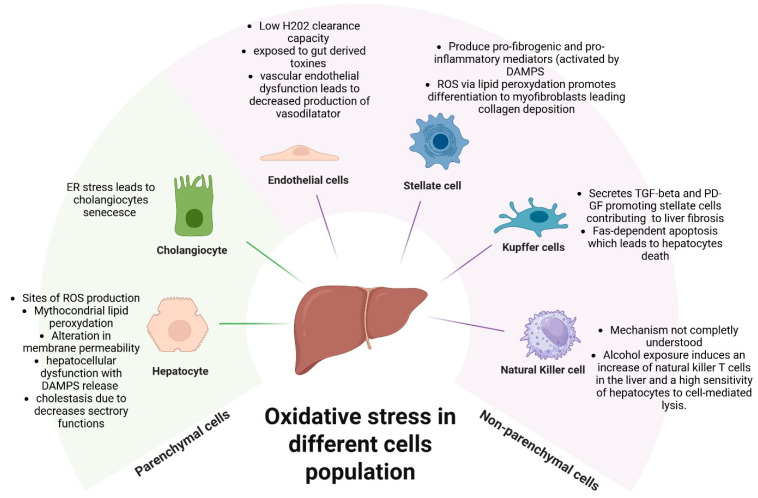
The susceptibility of hepatic cell types to oxidative stress. Created in BioRender. D’Arcangelo, F. (2026) https://BioRender.com (accessed on 3 March 2026).

Oxidative balance and homeostasis are crucial for physiological function. Reactive oxidant species (ROS) have dual roles as important signalling molecules regulating metabolism and cell survival in health, and driving protein, nuclear and lipid injury during pathogenic processes [31,32,33]. The liver, due to its role in metabolism, is particularly exposed to reactive oxygen species production, which causes potential damage when the oxidative balance is disrupted [34]. The unique cellular complexity of the liver means there are multiple different hepatic cell populations with distinct susceptibilities to oxidant stress (Figure 2). The hepatocyte population (80% of liver cells) performs essential functions, including detoxification, protein and lipid secretion, hormone and bile production [34,35,36]. Cholangiocytes, comprising 3–5% of liver cells, participate in bile synthesis, modification and excretion [37]. Non-parenchymal cells include liver sinusoidal endothelial cells (15–20% of liver cells), hepatic stellate cells (HSC), Kupffer cells (resident macrophages, KC), and natural killer (NK) cells [38,39], which influence the hepatic microenvironment and govern inflammatory cell recruitment, retention and fibrogenesis.

Reactive oxygen species (ROS) are produced mainly as a result of mitochondrial respiration, which generates superoxide radicals, although other sources of production have been identified as peroxisomes, xanthine oxidases, cytochrome P450 oxidases and the NADPH oxidases (NOXs) systems [40]. ROS can be subsequently categorised as unstable free radicals, such as superoxide radical and hydroxyl radical, which have potentially toxic effects, and stable non-radical oxidants like hydrogen peroxide and peroxynitrite. These non-radical oxidants have a fundamental role as messengers in different physiological pathways when oxidative balance is preserved [41]. However, NOX enzymes are considered responsible for ROS production, which enhances HSC and KC activation, leading to fibrosis progression via activation of the TGF-ß beta pathway [42,43,44]. Hepatocytes possess robust antioxidant mechanisms encompassing enzymatic components (superoxide dismutase, glutathione peroxidases, catalase, peroxiredoxins) and non-enzymatic molecules (glutathione, vitamins E, A, and C, ubiquinone) [45,46]. The enzymatic components act to metabolise ROS, while the non-enzymatic molecules mainly mitigate the damage caused by free radicals [47]. The nuclear factor erythroid 2-related factor 2 (NRF2)/antioxidant response element (ARE) pathway constitutes the master regulator of antioxidant defence. Under basal conditions, Kelch-like ECH-associated protein 1 (KEAP1) sequesters NRF2 in the cytoplasm, promoting its degradation. Oxidative stress modifies KEAP1 cysteine residues, thereby liberating NRF2 for nuclear translocation, where it activates transcription of cytoprotective genes such as glutathione S-transferases, NAD(P)H quinone oxidoreductase, SOD, CAT and GPX [48,49].

Oxidative stress contributes to the majority of liver pathologies [34]. In drug-induced liver injury, exemplified by acetaminophen toxicity, CYP450-mediated metabolism generates reactive intermediates that deplete glutathione stores, causing hepatocyte death [50,51,52]. Moreover, in metabolic dysfunction-associated steatotic liver disease (MASLD), lipid accumulation combined with mitochondrial dysfunction, lipid peroxidation, and oxidative DNA damage drives progression from simple steatosis to steatohepatitis (MASH) [53,54,55]. Chronic oxidative stress initiates a cascade of events promoting liver fibrosis [56,57]. Damaged hepatocytes release damage-associated molecular patterns and ROS that activate Kupffer cells, which secrete profibrogenic cytokines, including TGF-β [58]. This triggers hepatic stellate cell trans-differentiation into myofibroblasts, the principal collagen-producing cells in fibrotic liver [59]. The TGF-β/NOX axis amplifies this process through positive feedback mechanisms [44,60]. Paradoxically, while adaptive NRF2 activation protects against early liver disease, sustained NRF2 overexpression in established tumours promotes cancer cell proliferation and drug resistance [61,62].

## 4. Pathophysiology of Acute Alcohol-Associated Hepatitis

The cascade of events leading to the development of AH is incompletely described. These include events which are clearly attributable to arising oxidative stress and overwhelming of antioxidant protective mechanisms, and features which are independent of oxidative injury. These are considered in the sections below. Although most patients presenting with acute AH have a long history of alcohol use, it is unclear why some develop chronic disease while others develop an acute liver failure-type clinical presentation (even in the absence of signs of chronic damage), and others develop an acute chronic liver failure (ACLF) picture with high levels of systemic inflammatory response (SIRS). Genetic and epigenetic differences certainly play a role, with women—especially younger women—showing greater susceptibility to alcohol-induced injury compared to men [10,63]. The underlying cause is a dysfunction and death of hepatocytes due to alcohol metabolism in the liver, to which is added the role of chronic inflammation resulting from bacterial translocation caused by increased intestinal permeability, and the response to hepatocyte damage [10].

The majority of patients with AH develop the disease following episodes of major binge drinking. Preclinical mouse models using intragastric alcohol administration have demonstrated two distinct patterns of liver damage depending on the presence of binge alcohol use [64]. Mice with chronic alcohol exposure showed features of alcoholic steatohepatitis (ASH) characterised by macrophage-predominant inflammation and liver fibrosis, whereas mice exposed to binge drinking exhibited a shift toward neutrophil-predominant inflammation with clinical characteristics typical of AH [64,65]. One proposed theory to explain this phenomenon is that excessive alcohol consumption leads to massive hepatocyte damage with the release of damage-associated molecular patterns (DAMPs) and the development of SIRS [66]. This also occurs in the context of chronic alcohol consumption, which causes gut dysbiosis and translocation of pathogen-associated molecular patterns (PAMPs) from the gastrointestinal tract into the portal circulation. This translocation enhances the production of pro-inflammatory cytokines, exacerbating the SIRS and potentially leading to multi-organ failure [67]. Woodward Hopf et al. have analysed three different patterns of alcohol use: recurrent binge drinking, single binge drinking event and moderate alcohol consumption [68]. They observed increased levels of CYP2E1 expression and ADH mRNA, accompanied by higher concentrations of cytokine IL-1β in the recurrent binge drinking group [68]. Both ADH and CYP2E1 metabolise ethanol through oxidative pathways, and their up-regulation may further exacerbate oxidative stress associated with excessive alcohol intake. Moreover, increased levels of triglycerides were observed along with up-regulation of SREBP-1c mRNA and 4-hydroxynonenal (4-HNE), a marker of oxidative stress generated during ethanol metabolism [69], further supporting the presence of alcohol-induced oxidative damage in recurrent binge drinking mice [68].

### 4.1. Oxidative Stress and Cellular Dysfunction in Alcohol Liver Disease

Ethanol oxidation proceeds through two principal enzymatic pathways, which are used differentially depending on the nature, time course, and extent of alcohol consumption. Alcohol dehydrogenase (ADH) and the microsomal ethanol-oxidising system (MEOS), particularly cytochrome P450 2E1 (CYP2E1) [70,71]. ADH-mediated metabolism converts ethanol to acetaldehyde while reducing NAD+ to NADH, significantly altering the hepatic NAD+/NADH ratio and disrupting metabolic homeostasis [72,73]. This redox imbalance impairs fatty acid β-oxidation, promotes lipogenesis through SREBP-1c activation, and contributes to the characteristic steatosis observed in early ArLD [74]. CYP2E1 expression is induced 4- to 10-fold by chronic alcohol consumption and represents a major source of hepatic ROS. This enzyme exhibits loose coupling between substrate oxidation and electron transfer, resulting in substantial electron leakage and generation of superoxide anions, hydrogen peroxide, and hydroxyl radicals [6,75]. Furthermore, CYP2E1 displays elevated expression not only in hepatocytes but also in adipose tissue during chronic ethanol exposure, contributing to systemic oxidative stress through increased lipid peroxidation products, including 4-hydroxynonenal (4-HNE) and decreased glutathione ratios [3].

NADPH oxidases (NOXs) constitute another critical source of ROS during ethanol metabolism in the liver. Among the seven NOX family members, NOX1, NOX2, and NOX4 are predominantly expressed in liver cells [76]. NOX4 expression is significantly increased in the mitochondrial fraction following chronic alcohol exposure, establishing a direct link between NOX activity and mitochondrial oxidative stress [77]. NOX2, primarily expressed in Kupffer cells and infiltrating neutrophils, generates ROS in response to lipopolysaccharide (LPS) stimulation, linking gut-derived endotoxin exposure and ROS generation, leading to inflammatory liver injury [78]. Studies using NOX1/NOX4 inhibitors such as GKT137831 have demonstrated attenuation of hepatic fibrosis and apoptosis, validating these enzymes as therapeutic targets [79,80]. Hepatocytes possess robust antioxidant defence mechanisms comprising enzymatic components, including superoxide dismutase (SOD), glutathione peroxidases (GPX), catalase (CAT), peroxiredoxins (PRX), and thioredoxins (TRX), as well as non-enzymatic antioxidants such as glutathione (GSH), vitamin E, vitamin C, and coenzyme Q10 [81]. The NRF2/Kelch-like ECH-associated protein 1 (KEAP1) pathway represents the master regulator of cellular antioxidant defence; however, alcohol consumption impairs its signalling, leading to GSH depletion and creating a pro-oxidant environment that perpetuates hepatocyte injury [48,49].

ROS-mediated injury is particularly evident in mitochondria, as they serve as both major sources and targets of ROS in hepatocytes. The electron transport chain, particularly complexes I and III, generates superoxide radicals under normal physiological conditions [82]. Chronic alcohol exposure amplifies mitochondrial ROS production through multiple mechanisms, including electron transport chain dysfunction, CYP2E1 induction, and impaired mitochondrial DNA (mtDNA) repair [82,83]. The resulting oxidative damage compromises mitochondrial membrane integrity, leading to mitochondrial permeability transition (MPT) and subsequent release of pro-apoptotic factors, including cytochrome c and increased capsase-3 [84,85]. Moreover, mitochondrial DNA (mtDNA) is particularly vulnerable to oxidative damage due to its proximity to the electron transport chain and limited repair mechanisms [86]. Oxidatively damaged mtDNA released into the cytoplasm acts as a DAMP, activating the NLRP3 inflammasome through TLR9 signalling and triggering the STING (stimulator of interferon genes) pathway to induce type I interferon responses [87]; hence, MtDNA-mediated inflammation represents a critical link between mitochondrial oxidative stress and hepatic inflammatory injury [88].

Recently, endoplasmic reticulum (ER) stress has been recognised to play a role in hepatocyte dysfunction since redox homeostasis is essential for proper protein folding [89]. Chronic alcohol exposure activates the unfolded protein response (UPR) as evidenced by increased expression of glucose-regulated proteins GRP78 and GRP94, CHOP, and caspase-12, which contributes to hepatic steatosis through activation of SREBP-1c and SREBP-2 transcription factors that promote lipogenesis [74]. This pathway seems to enhance the innate immune response, enhancing hepatic inflammation and cell damage [90]. Oxidative stress activates multiple regulated cell death pathways in hepatocytes. Apoptosis, mediated through both intrinsic (mitochondrial) and extrinsic (death receptor) pathways, involves cytochrome c release, apoptosome formation with APAF1, and caspase cascade activation [91]. Subsequently, the balance between pro-apoptotic (BAX, BAK) and anti-apoptotic (Bcl-2, Bcl-xL) proteins determines hepatocyte susceptibility to apoptotic stimuli [11]. This mechanism is not the only one involved in hepatocyte death, with Ferroptosis, a recently characterised iron-dependent cell death mechanism, increasingly recognised in ArLD pathogenesis [92]. Ferroptosis is characterised by overwhelming lipid peroxidation in an iron-dependent manner, leading to membrane rupture distinct from apoptotic cell shrinkage [93]. GSH depletion and GPX4 inactivation remove critical antioxidant defences against lipid peroxidation, rendering hepatocytes susceptible to ferroptotic death [94]. Finally, Necroptosis, a programmemed form of necrosis mediated by RIPK1/RIPK3/MLKL signalling, and pyroptosis, involving NLRP3 inflammasome activation and gasdermin D-mediated membrane pore formation, represent additional death pathways activated by oxidative stress in ArLD [95].

ROS and lipid peroxidation products also directly activate hepatic stellate cells (HSCs), inducing their transformation from quiescent vitamin A-storing cells to proliferative myofibroblasts [58]. Activated HSCs lose their vitamin A content, express α-smooth muscle actin (α-SMA), and produce excessive extracellular matrix (ECM) components, including type I collagen, fibronectin, and proteoglycans [56]. TGF-β, the most potent pro-fibrogenic cytokine, activates HSCs through SMAD2/3-dependent pathways and directly induces COL1A1 and COL1A2 gene transcription [96]. ROS amplify TGF-β signalling, while TGF-β reciprocally upregulates NOX4 expression, creating a self-perpetuating fibrogenic cycle [60]. PDGF released by activated HSCs increases tissue inhibitor of metalloproteinase (TIMP) expression, inhibiting collagenase activity and promoting ECM accumulation [97].

ROS is also considered an indicator and driver of senescence, which is defined as a state of cell cycle blockage in which apoptosis is arrested [98,99]. Rodrigo-Torres et al. performed RNA sequencing and bioinformatics analysis in patients affected by AH [100]. Their longitudinal analysis revealed a reduction in senescence-related gene expression in the liver during clinical resolution of AH after acute injury [100]. Bioinformatic profiling further uncovered a transcriptomic pattern involving senescence, apoptosis, and proliferation that becomes progressively more disrupted as ALD severity increases, with marked upregulation of senescence markers in AH. Although transcriptomic data suggest partial reversibility of this response during recovery, senescence-associated proteins remained elevated after 28 days, indicating persistence of senescent cells [100]. The authors’ hypothesis that senescence amplifies mitochondrial dysfunction in alcohol-related injury [100].

### 4.2. SIRS

In patients who develop AH, inflammatory mediators play a central role in driving systemic inflammation alongside parenchymal cell damage. As previously discussed, when hepatocyte function becomes severely compromised due to excessive alcohol intake, PAMPs crossing the intestinal barrier amplify cellular injury. This inflammatory cascade involves TNF-α signalling and increased expression of multiple chemokines, including IL-8, CXCL-5, Gro-γ, CXCL-6, IL-1, osteopontin, and MCP1/CCL2 [101,102,103]. Recently, IL-8 levels have been reported to be particularly elevated in patients with sAH [104].

Beyond the inflammatory component, impaired hepatic regeneration likely represents another critical factor underlying liver decompensation in AH patients. Examination of explanted livers from AH patients after liver transplantation showed that individuals resistant to medical management exhibited decreased levels of regeneration-associated cytokines (tumour necrosis factor α and interleukin-6), along with no markers of hepatocyte proliferation [105]. Furthermore, AH patients frequently display marked proliferation of liver progenitor cells (LPCs), manifesting as a ductular reaction. However, in this context, LPCs are not able to mature into functional hepatocytes, and their presence correlates directly with disease severity and early mortality risk in this patient population [106].

Neutrophil infiltration represents a hallmark of AH and is tightly integrated with oxidative stress through bidirectional signalling. In this context, the innate immune system, particularly Kupffer cells and neutrophils, orchestrates the inflammatory response that amplifies hepatic injury while also participating in tissue repair [7]. The degree of hepatic neutrophil infiltration correlates with MELD score and predicts mortality, as per the neutrophil-to-lymphocyte ratio, which is also acknowledged as a prognostic biomarker in severe AH [7]. Neutrophil recruitment involves a coordinated chemokine response: activated Kupffer cells release CCL2 and CXCL2, hepatic sinusoidal endothelial cells (HSECs) upregulate CXCL1 and CXCL8, and activated stellate cells contribute additional CXCL1 and CXCL8. The HSEC are particularly sensitive to ROS and oxidants generated by KC activation, which lead to enhanced chemokine-dependent neutrophil recruitment across the sinusoids [103,107,108,109]. The IL-17 pathway plays a central role in amplifying neutrophil recruitment. Th17 cells recruited by CCL2 release IL-17, which activates stellate cells to produce neutrophil-attracting chemokines [110]. Moreover, the inflammatory response and oxidative stress seem to be related through IL-6 trans-signalling by the IL-6/sIL-6R complex, which regulates neutrophilic infiltration in AH and enhances ROS generation through p47phox [111,112]. MicroRNA-223 (miR-223), which normally protects against oxidative stress by inhibiting the IL-6-p47phox pathway, is downregulated in AH neutrophils, increasing their susceptibility to oxidative damage [113]. Moreover, circulating neutrophils in AH display baseline activation predisposing to spontaneous NETosis (neutrophil extracellular trap formation) and release of ROS and proteases, all of which amplify local tissue damage [7]. Recently, Schnabl et al. [114] applied tandem mass tag (TMT)-based proteomic profiling to examine faecal proteins in patients with severe alcohol-associated hepatitis (sAH), comparing them with healthy controls and individuals with alcohol use disorder. Their analysis showed that the proteins altered in sAH are predominantly associated with neutrophil granules and the neutrophil degranulation pathway. Myeloperoxidase (MPO), a key granule-derived neutrophil marker, was strongly linked to disease severity and served as a predictor of 60-day mortality. In an independent validation cohort, the authors verified that faecal MPO concentrations were associated with short-term (60-day) survival. These findings underscore the central involvement of neutrophils in the pathogenesis of sAH [114].

Neutrophils in AH exhibit phenotypic and functional abnormalities in addition to their changes in basal activation. Upon density gradient centrifugation, neutrophils separate into high-density neutrophils (HDNs) with classical morphology and low-density neutrophils (LDNs) with altered functional capacity [115]. An increase in LDN populations has been observed in AH patients, with these cells having an impaired response to ROS and LPS, leading to a reduction in phagocytosis [116,117]. However, the potential pleiotropic roles of neutrophils make them a challenging target for potential treatments. As an example, neutrophils in AH promote hepatocyte regeneration via secretion of hepatocyte growth factor (HGF) [117], and so depletion of neutrophils is detrimental in late stages of injury. This may be linked to the role of neutrophil-derived oxidants in promoting macrophage polarisation to the restorative phenotype, which drives organ healing [114,118].

### 4.3. Gut–Liver Axis Dysfunction

Chronic alcohol consumption disrupts intestinal barrier integrity through iNOS-dependent ROS production in enterocytes and alterations in tight junction proteins [119]. Alcohol-induced dysbiosis, characterised by decreased Faecalibacterium prausnitzii (butyrate producers) and increased proteobacteria, further compromises barrier function [120,121]. The resulting translocation of lipopolysaccharide (LPS) and other pathogen-associated molecular patterns (PAMPs) to the liver via the portal circulation activates Kupffer cells through TLR4 signalling [122]. NADPH oxidase-derived ROS in Kupffer cells mediate NF-κB activation and subsequent cytokine production, establishing another direct link between oxidative stress and inflammation [123]. In fact, NF-κB leads to an increase in TNF-α, which decreases the activity of the PPAR gamma gene, resulting in an increase in beta oxidation with consequent steatosis [124]. Moreover, TNF-α stimulates Kupffer cells to produce IL-8/CXCL-8, which are involved in neutrophil mobilisation and correlate with patient survival in AH [103,125]. Alcohol-induced dysbiosis has been reported by several authors who have examined the microbiome of patients with AH, revealing increased prevalence of pathogenic taxa, such as Enterobacteriaceae, Streptococcaceae, and Enterococcus [119]. Fouts and Bernd Schnabl et al. [126] observed in patients with AH an increase in E. faecalis, which correlated with liver disease severity and with mortality in patients with AH [126].

## 5. Therapies—Current and Experimental

Despite significant advances in understanding ArLD pathogenesis, effective pharmacological therapies remain limited and there is no FDA-approved drug treatment specifically for acute-associated hepatitis. Current treatments focus primarily on supportive care (e.g., nutritional and decompensation of cirrhosis management) and alcohol abstinence, with corticosteroids serving as the only established pharmacological intervention for severe AH. However, several emerging therapies targeting oxidative stress, inflammation, and hepatic regeneration are under investigation. These are summarised in Table 1. Corticosteroids remain the first-line pharmacological treatment for severe AH (Maddrey Discriminant Function ≥ 32 or MELD > 20, with a significant survival benefit in patients with MELD score between 25 and 39, with no benefit in patients with MELD > 51 [13,127]. A recent meta-analysis of 52 randomised controlled trials (5121 participants) demonstrated that corticosteroids reduce 28-day mortality compared to placebo (RR 0.62; 95% CI 0.41–0.95) [128]. However, available data suggest this does not extend survival beyond 28 days and concerns regarding increased infection susceptibility persist [129,130]. Concern around the prevalence of bacterial and fungal infection, for patients on steroid therapy, is around 16% and associated with a consequent high risk of mortality [131].

Among antioxidant therapies, N-acetylcysteine (NAC), which is the treatment of choice for paracetamol hepatotoxicity, restores hepatic glutathione stores and reduces endoplasmic reticulum (ER) stress [132]. However, NAC monotherapy did not demonstrate survival benefit in AH, whereas the combination therapy with corticosteroids reduced 28-day mortality compared to corticosteroids alone (RR 0.35; 95% CI 0.16–0.78), though this benefit was not maintained out to six months [128]. S-adenosyl-L-methionine (SAMe), which plays a role in GSH synthesis, increasing hepatic glutathione content, was also suggested as a promising antioxidant therapy. However, patients treated with SAMe exhibited similar biochemical and histological scores when compared with placebo in randomised controlled trials [133,134]. Other agents look more promising, with Metadoxine, which accelerates acetaldehyde clearance and possesses antioxidant properties, showing benefit both at 28 days (RR 0.47; 95% CI 0.25–0.90) and 90 days when combined with corticosteroids in a recent meta-analysis [128]. Vitamin E, a lipid-soluble antioxidant, has demonstrated efficacy in NAFLD/NASH with reductions in ALT and AST levels and is recommended by AASLD guidelines [135] for MASLD patients. Although vitamin E nano-emulsions targeting CYP2E1-induced ER stress showed protective effects [136,137], studies in patients with mild to moderate alcohol-related hepatitis suggest limited benefit [138]. Finally, natural products with antioxidant properties have demonstrated hepatoprotective effects in preclinical studies [139]. In this context, curcumin from turmeric reduces MDA and inhibits NF-κB activation; Resveratrol from grapes enhances antioxidant enzyme activities and modulates inflammatory pathways; and Green tea catechins increase GSH and SOD activities while reducing MDA in chronic alcohol-fed animals, though rigorous clinical trials are needed to establish efficacy and safety [140,141,142].

The gut–liver axis also presents new targets for therapy. Gut dysbiosis is linked to hepatocyte and Kupffer cell activation, NADPH oxidase activation and mitochondrial dysfunction, and so restoration of microbial homeostasis has obvious benefit. In this context, faecal microbiota transplantation (FMT) addresses alcohol-induced dysbiosis and has emerged as a promising approach [10]. A randomised trial comparing FMT to prednisolone in severe AH demonstrated a reduction in 90-day mortality RR 0.58 (95% CI 0.37–0.92) [128]. A possible explanation could be that FMT restored microbial diversity and reduced intestinal permeability in treated patients. There is also evidence that healthy microbial metabolites, such as short-chain fatty acids and alpha linolenic acid, have antioxidant properties that are beneficial in alcohol-related injury [143]. However, standardisation of donor screening, preparation protocols, and delivery methods remains challenging [2]. Currently, results are awaited from several clinical trials targeting antibiotics and probiotic use, FMT, and gut barrier function [7].

Among therapies targeting hepatic regeneration, Granulocyte colony-stimulating factor (G-CSF), which mobilises bone marrow stem cells and promotes hepatocyte proliferation, has shown promising results in combination with pentoxifylline vs. pentoxifylline alone at 28-day mortality and at 90-day mortality in combination with steroids vs. steroids alone [128,144]. This could be linked to the recruitment of neutrophils and subsequent polarisation of restorative macrophages as discussed previously. Certainly, in acute paracetamol toxicity, administration of GCSF has been linked to a reduction in oxidative damage [145] but this may not be the case in acute alcohol injury. Attempting to modify the inflammatory consequences of tissue injury in alcohol exposure by modifying cytokine function has yielded disappointing results [146]. Targeting IL-22, a cytokine with hepatoprotective and regenerative properties, the IL-22 agonist F-652 showed safety and efficacy signals in a phase-2 dose-escalating study, resulting in inflammatory cytokine down-regulation and improvements in MELD scores and bilirubin levels [147]. The ISAIAH trial evaluated canakinumab, an anti-IL-1β monoclonal antibody, in AH patients with mDF ≥ 32 and MELD ≤ 27. Every patient underwent liver biopsy before and after 28 days of treatment, despite histological improvement in mononuclear infiltrate, no clinical benefit was observed [148]. Similarly, Canakinumab (IL-1 β receptor antagonist) has been associated with histological improvement without improving disease severity score and clinical outcomes [148]. In fact, the randomised trial of anakinra plus zinc vs. prednisone was stopped early due to worsening MELD score and uncontrolled infection events [149]. Finally, conflicting results have been reported in clinical trials investigating the role of G-CSF in stimulating neutrophil migration from bone marrow without a difference in survival or infection risk in European and American cohorts [150,151]. As described earlier, the pleiotropic role of neutrophils, which are involved in both inflammatory and repair pathways, makes it difficult to target their effects in a single, predictable way.

Molecular-targeted approaches under investigation include NOX inhibitors such as GKT137831, which attenuated fibrosis and apoptosis in preclinical models [79]. Mitochondria-targeted antioxidants, including MitoQ concentrate, an ubiquinone derivative, which accumulates within mitochondria acting as an antioxidant, were able to prevent lipid peroxidation in an experimental model of ALD [152]. Strategies to limit hepatic neutrophil infiltration through blockade of adhesion molecules (ICAM-1, E-selectin, CD44) and chemotaxis are limited [153,154,155]. Recent evidence has explored the link between mitochondrial dysfunction and damage to reveal new pathways for potential treatments, including MicroRNA-based therapies [156]. Recently, Rodríguez-Agudo et al. [156] observed an increased expression of miR-873-5p in hepatocytes of ArLD patients. This is a potential key regulator of NAD metabolism and SIRT1 deacetylase activity [141]. The authors were able to restore SIRT1 expression and bile acid homeostasis by utilising anti-miR-873-5p, thereby reducing mitochondrial ROS, ER stress, and hepatocyte death in experimental models. Rodrigo-Torres et al. [100], using transcriptomic datasets together with bioinformatic approaches, recently documented that cellular senescence undergoes dynamic modulation in the context of injury. During the recovery phase of AH, senescence-associated markers in the liver decline compared to their levels at the moment of injury. The analyses also revealed a broader gene-expression program involving senescence, apoptosis, and cell proliferation, which becomes increasingly disrupted as ArLD progresses. Notably, senescence-related genes are strongly elevated in AH. These insights indicate that therapeutic strategies aimed at senescence pathways in the early stages of ArLD may help reduce the intensity of acute disease flares [100].

Another line of research has been investigated by Goikoetxea-Usandizaga et al. [157] who downregulated the methylation-controlled J protein (MCJ), also known as DnaJC15, which is an endogenous negative regulator of mitochondrial activity. The authors found reduced survival in whole-body knockout of MCJ after alcohol exposure, with observed increased hepatic steatosis as a result of increased lipid peroxidation and reduced mitochondrial function, with no difference in terms of immune infiltrate when compared with normal mice. Moreover, augmented intestinal permeability and translocation of bacterial products were reported along with hyperglycaemia due to pancreatic beta-cell dysfunction. Promising results have been observed using MCJ siRNA with reduced lipid deposit, oxidative stress and inflammatory response [157]. Finally, Artru et al., [158] explored the potential role of endogenous bioactive liver mediators through untargeted lipidomics from a large cohort of patients with sAH in order to identify lipid mediators involved in the pathogenesis of sAH. The authors observed high levels of acylcarnitine, a long-chain fatty acid considered a mitochondrial dysfunction marker, in the plasma of patients with sAH. Moreover, Acylcarnitines were found to correlate with MELD, pro-inflammatory cytokine levels, and hepatocyte ballooning on histology [158]. Experimental therapies are summarised in Table 1.

**Table 1:** Current and experimental treatments for AH.

Treatment	Indication	Mechanism of Action	Pro/Cons	Clinical/Pre-Clinical Status	Approved by FDA
Corticosteroids	Maddrey Discriminant Function ≥ 32 or MELD >20 [12,127]	Suppress the immune/inflammatory response triggered by alcohol, reducing pro-inflammatory cytokines (like TNF-α), inhibiting neutrophil activation.	Reduced 28-day mortality but not long-term. Concern of infectious risk [127,128,129,130,131]	Clinical	Approved by FDA but not for this indication
Antioxidants					
N-acetylcysteine (NAC)		Restores hepatic glutathione stores and reduces endoplasmic reticulum (ER) stress [132]	Combination therapy with corticosteroids reduced 28-day mortality compared to corticosteroids alone, though this benefit was not conferred out to 6 months [131]	Clinical	Approved by FDA but not for this indication
S-adenosyl-L-methionine (SAMe)		GSH synthesis increases hepatic glutathione content [133]	Combination therapy with corticosteroids reduced 28-day mortality compared to corticosteroids alone but the benefit was not conferred out to 6 months [133,134]	Clinical	Not approved
Metadoxine		Accelerates acetaldehyde clearance	Showed benefit both at 28 days (RR 0.47; 95% CI 0.25–0.90) and 90 days when combined with corticosteroids in a recent meta-analysis [128]	Clinical	Not approved
Gut–Liver axis target					
Faecal microbiota transplantation (FMT)		The possible mechanism of action is to restore microbial diversity and reduce intestinal permeability	A randomised trial comparing FMT to prednisolone in severe AH demonstrated a reduction in 90-day mortality [128]. However, standardisation of donor screening, preparation protocols, and delivery methods remains challenging.	Clinical	Approved by FDA but not for this indication
Hepatic regeneration target					
Granulocyte colony-stimulating factor (G-CSF)		Mobilises bone marrow stem cells and promotes hepatocyte proliferation	Shown conflicting results. Promising results in combination with pentoxifylline vs. pentoxifylline alone at 28-day mortality and at 90-day mortality in combination with steroids vs. steroids alone [128,144]. No benefits reported among subjects with AH who received pegfilgrastim + prednisolone compared with subjects receiving prednisolone alone [153].	Clinical	Approved by FDA but not for this indication
Direct cytokine antagonism					
IL-22 agonist F-652		Hepatoprotective and regenerative properties	Showed safety and efficacy signals in a phase-2 dose-escalating study resulting in inflammatory cytokine down-regulation and improvements in MELD scores and bilirubin levels [147]	Clinical	Not approved
Canakinumab		Anti-IL-1β monoclonal antibody	ISAIAH trial reported histological improvement in mononuclear infiltrate; however, no clinical benefit was observed [148,149] randomised trial of anakinra plus zinc vs. prednisone was stopped early due to worsening MELD score and uncontrolled infection events [149]	Clinical	Approved by FDA but not for this indication
Molecular target					
GKT137831		NOX inhibitors	Attenuated fibrosis and apoptosis in preclinical models [82]	Pre-clinical	Not applicable
MitoQ concentrate		Mitochondria-targeted antioxidants	Prevent lipid peroxidation in an experimental model of ALD [152]	Pre-clinical	Not applicable
Hepatic Neutrophil infiltration target					
blockade of adhesion molecules (ICAM-1, E-selectin, CD44)		Blockade of adhesion molecules (ICAM-1, E-selectin, CD44)	Difficult to target due to their pleiotropic effects [114]	Pre-clinical	Not applicable
IL-6 trans-signalling (TS)		IL-6 trans-signalling (TS) enhances STAT3-dependent gene expression in hepatocytes, which sustains neutrophilic inflammation in alcoholic hepatitis [158]	Blocking IL-6 TS could be a new therapeutic target [158]	Pre-clinical	Not applicable
MicroRNA-based therapy					
Anti-miR-873-5p		Potential key regulator of NAD metabolism and SIRT1 deacetylase activity [100]	Restore SIRT1 expression and bile acid homeostasis, reducing mitochondrial ROS, ER stress, and hepatocyte death in experimental models [157]	Pre-clinical	Not applicable
MCJ siRNA		Endogenous negative regulator of mitochondrial activity	Reduced lipid deposit, oxidative stress and inflammatory response [157]	Pre-clinical	Not applicable
Endogenous bioactive liver mediators					
Anti-Acylcarnitines		Acylcarnitine is a long-chain fatty acid considered a mitochondrial dysfunction marker	Acylcarnitines were found to correlate with MELD, pro-inflammatory cytokine levels, and hepatocyte ballooning on histology [158]	Pre-clinical	Not applicable

Summary of current and experimental treatment strategies used for managing alcoholic hepatitis, where therapies are divided on the basis of their mode of action and stage of clinical development is indicated.

## 6. Conclusions

Oxidative stress and inflammation represent intertwined pathogenic mechanisms in ArLD and AH that perpetuate hepatocyte injury and drive disease progression. The molecular crosstalk between ROS generation, mitochondrial dysfunction, ER stress, inflammasome activation, and multiple cell death pathways creates a complex network that defies simple therapeutic intervention. Neutrophils, while contributing to tissue damage through ROS and protease release, also participate in hepatic repair, necessitating meticulous therapeutic approaches. Current pharmacological options remain limited to corticosteroids for sAH and liver transplantation in selected patients. However, understanding of the contribution of the cellular balance of oxidant signalling in alcohol-related disease does raise the possibility of novel therapies. However, despite recent advances in our pathophysiological understanding, significant gaps in the literature persist. In particular, it remains unclear how alcohol exposure can lead to such a wide spectrum of clinical manifestations, ranging from simple steatosis to chronic liver disease and severe conditions such as acute-on-chronic liver failure and acute hepatitis. Understanding interindividual and gender-specific variation in response to similar alcohol exposure, the impact of comorbidities such as steatotic liver disease and the interplay between nutritional status, genetic background and microbiome constituents will be important for future developments in this space. Novel insights are expected to arise from pre-clinical studies currently investigating different potential new therapies. Molecular-targeted approaches, including NOX inhibitors and drugs to correct mitochondrial dysfunction, represent an exciting frontier, as they address upstream mechanisms of hepatocyte injury rather than downstream inflammatory consequences alone.

The integration of transcriptomic, lipidomic, and bioinformatic approaches into clinical trial design holds particular promise for uncovering novel therapeutic targets and accelerating the translation of preclinical findings into personalised clinical practice. Finally, we believe that the key challenge moving forward will be to integrate these diverse molecular pathways into a unified and clinically applicable therapeutic strategy, or alternatively, to identify the most appropriate target based on individual patient characteristics in the era of precision medicine. In this context, novel biochemical and clinical biomarkers may prove valuable in the future, potentially helping clinicians to tailor treatments to individual patients, beyond the use of conventional prognostic scores based solely on clinical features.

## Data Availability

Please add the corresponding content.
